# Blockade of PD-1, PD-L1, and TIM-3 Altered Distinct Immune- and Cancer-Related Signaling Pathways in the Transcriptome of Human Breast Cancer Explants

**DOI:** 10.3390/genes11060703

**Published:** 2020-06-25

**Authors:** Reem Saleh, Salman M. Toor, Dana Al-Ali, Varun Sasidharan Nair, Eyad Elkord

**Affiliations:** 1 Cancer Research Center, Qatar Biomedical Research Institute (QBRI), Hamad Bin Khalifa University (HBKU), Qatar Foundation (QF), Doha 34110, Qatar; rsaleh@hbku.edu.qa (R.S.); mstoor@hbku.edu.qa (S.M.T.); vsnair@hbku.edu.qa (V.S.N.); 2 Department of Medicine, Weil Cornell Medicine-Qatar, Doha 24144, Qatar; dka2003@qatar-med.cornell.edu

**Keywords:** primary breast cancer, transcriptomic profiling, immune checkpoint inhibitors, immune responses

## Abstract

Immune checkpoint inhibitors (ICIs) are yet to have a major advantage over conventional therapies, as only a fraction of patients benefit from the currently approved ICIs and their response rates remain low. We investigated the effects of different ICIs—anti-programmed cell death protein 1 (PD-1), anti-programmed death ligand-1 (PD-L1), and anti-T cell immunoglobulin and mucin-domain containing-3 (TIM-3)—on human primary breast cancer explant cultures using RNA-Seq. Transcriptomic data revealed that PD-1, PD-L1, and TIM-3 blockade follow unique mechanisms by upregulating or downregulating distinct pathways, but they collectively enhance immune responses and suppress cancer-related pathways to exert anti-tumorigenic effects. We also found that these ICIs upregulated the expression of other IC genes, suggesting that blocking one IC can upregulate alternative ICs, potentially giving rise to compensatory mechanisms by which tumor cells evade anti-tumor immunity. Overall, the transcriptomic data revealed some unique mechanisms of the action of monoclonal antibodies (mAbs) targeting PD-1, PD-L1, and TIM-3 in human breast cancer explants. However, further investigations and functional studies are warranted to validate these findings.

## 1. Introduction

Despite recent declines in mortality rates, breast cancer remains the most frequently diagnosed malignancy in females worldwide, and it is among the leading causes of cancer-related deaths [1,2]. The devastating incidence and burden of breast cancer can be attributed to the biological heterogeneity of breast tumors, which makes screening and treatment selection challenging. Current treatments for breast cancer primarily include tumor removal by surgery, radiotherapies, or systematic therapies using hormone receptor-targeted therapies or small molecule inhibitors combined with chemotherapy [3].

Immune checkpoints (ICs) alter T cell responses by regulating their activation pathways [4]. Tumors can exploit these pathways by upregulating inhibitory ICs and their ligands in the tumor microenvironment (TME) to evade immunosurveillance [5]. Therefore, monoclonal antibodies (mAbs) that target inhibitory ICs have provided immense breakthroughs in cancer therapeutics [6]. Immune checkpoint inhibitors (ICIs) targeting programmed cell death protein 1 (PD-1) and cytotoxic T-lymphocyte-associated protein 4 (CTLA-4) have been at the forefront of all anti-cancer immunotherapies and are currently approved for the treatment of various cancers, including those of melanoma, lung, bladder, gastric, cervix, head, neck, Merkel cell, hepatocellular, cutaneous squamous cell cancers, classic Hodgkin’s lymphoma, and B-cell lymphoma [7]. Moreover, hyper mutated tumors that present with DNA mismatch repair deficiency (dMMR) and microsatellite-instability (MSI-H) have shown better responses to ICIs and led to the approval of anti-PD-1 therapy for treating any advanced or recurrent MSI-H/dMMR solid tumors that continue to progress after conventional chemotherapies [8]. However, these mutations are rarely detected in breast tumors, and the mainstream use of ICIs remains debatable due to low response rates and frequent reports of immune-related adverse events in some patients [6,9].

Cancer immunotherapy in the form of ICIs is yet to have a major impact on breast cancer treatment. The presence of immune cell infiltrates, including tumor-infiltrating lymphocytes (TILs), in triple negative breast cancer (TNBC) with PD-1 or programmed death ligand-1 (PD-L1) expression suggest that ICIs could be effective in treating TNBC and enhancing anti-tumor immunity [10,11]. Higher levels of TILs were detected in tumor samples from patients with human epidermal growth factor receptor 2 (HER2)-positive breast cancer and TNBC than those with hormone-dependent subtypes [12]. However, effects of ICIs on other types of breast cancer positive for hormone receptor expression warrant further investigations. Atezolizumab (anti-PD-L1), in combination with paclitaxel, was recently approved by the U.S. Food and Drug Administration (FDA) for PD-L1-positive, unresectable, locally advanced, or metastatic TNBC, since it has shown prolonged progression-free survival in these patients [13]. In addition, emerging ICIs for breast cancer are currently under pre-clinical and clinical development [14].

ICs such as T cell immunoglobulin and mucin-domain containing-3 (TIM-3), lymphocyte activation gene-3 (LAG-3), T cell immunoglobulin and ITIM domain (TIGIT), and V-domain Ig suppressor of T cell activation (VISTA) have emerged as novel therapeutic targets, and their inhibition has shown promising results in various preclinical and clinical studies [5,15]. These novel targets and the identification of methods to discover predictive biomarkers of therapy response were the fruit of extensive ongoing research that has benefited from advanced sequencing technologies. However, there is a dire need to understand the effects of different ICIs on immune cell populations and the molecular pathways they regulate to potentially uncover resistance mechanisms and to improve response rates in cancer patients.

In this study, we investigated the effects of different ICIs on human primary breast cancer (PBC), with hormone-dependent subtypes, using explant cultures. We cultured breast tumor tissues in the presence or absence of anti-PD-1, anti-TIM-3 or anti-PD-L1 mAb, and then we performed investigations using flow cytometric analyses and RNA sequencing (RNA-Seq). We investigated the expression levels of different ICs and T regulatory cell (Treg)-related markers on T cells, and we performed transcriptomic analyses to uncover potential mechanisms of action of ICIs in targeting immunosuppressive factors and genes/signaling pathways that may contribute to low response rates and resistance to the different ICIs. Our transcriptomic data revealed unique mechanisms of action of these three ICIs, which have the potential to predict therapy response in breast cancer patients.

## 2. Materials and Methods

### 2.1. Sample Collection and Processing

Tumor tissues (TT) were obtained from 2 treatment-naïve primary breast cancer (PBC) patients who provided written informed consent prior to sample donation. Patient 1 presented with stage II, while Patient 2 presented with stage III. Both patients presented with estrogen receptor (ER^+^), progesterone receptor (PR^+^), HER2^−^, and poorly differentiated tumors.

Tumor tissues were cut into small pieces and frozen in 1 mL of freezing medium [10% dimethyl sulphoxide (DMSO; Sigma-Aldrich, St. Louis, MO, USA), 50% fetal calf serum (FCS; Hyclone, GE Healthcare Life Sciences, Logan, UT, USA) and 40% RPMI-1640 medium (Life Technologies, Grand Island, NY, USA)] and stored for subsequent analyses, as previously described [16].

This study was executed under ethical approval from Qatar Biomedical Research Institute, Doha, Qatar (Protocol no. 2017-006), and all experiments were executed in accordance with applicable guidelines and regulations.

### 2.2. Explant Culture and Treatment Conditions

Frozen tissue specimens were thawed and washed with phosphate-buffered saline (PBS). Tissue specimens were then mechanically cut into small pieces (~2–4 mm) using a surgical scalpel. Tissue pieces were then cultured in a 6-well tissue culture-treated plate in complete media (RPMI-1640 media (Life Technologies) supplemented with 10% FCS (Hyclone) and 1% penicillin/streptomycin (Hyclone)) and 600 international units (IU) of interleukin-2 (IL-2, PeproTech, Hamburg, Germany) in the presence or absence of different immune checkpoint inhibitors, including 2 µg/mL of anti-PD-1 mAb (pembrolizumab; Merck, Branchburg, NJ, USA), 0.5 µg/mL of anti-PD-L1 mAb (atezolizumab, BioVision Inc., Milpitas, CA, USA), or 10 µg/mL of anti-TIM-3 (functional grade, Biolegend, San Diego, CA, USA). The dosage used of mAbs was based on titration that was previously performed [17,18], as previously published [19,20,21,22]. The treatment with ICIs was done on day 0. Explant cultures were maintained for up to 25 days, and media/IL-2 were replenished every 48 h. Non-adherent cells [referred to as tumor-infiltrating immune cells (TIICs)] were collected from the non-treated and treated wells on different days for flow cytometric and molecular investigations. Aggregates and debris were removed from cell suspensions by first passing them through a 100 µm cell strainer and then by washing with PBS before staining for flow cytometric analyses on day 9, and RNA extraction was performed on day 25. Cell viability was checked at regular intervals, and day 9 (an early time point) was selected to check cell proliferation and determine the percentage of TILs in the culture using flow cytometric analyses. A later time point (day 25) was selected for nucleic acid extractions and downstream RNA sequencing to allow cells to proliferate for the adequate yield of nucleic acids.

### 2.3. Multi-Parametric Flow Cytometry

Cells were stained as previously described [16]. Briefly, Fc receptors (FcR) were first blocked using FcR Blocking Reagent (Miltenyi Biotech, Bergisch Gladbach, Germany), and 7-AAD viability dye (eBioscience, San Diego, CA, USA) was used to gate live cells. Cells were stained with cell surface antibodies against CD3-Alexa Fluor 700 (clone UCHT-1; BD Biosciences, Oxford, UK), CD4-phycoerythrin (clone RPA-T4; BD Biosciences), CD45-fluorescein isothiocyanate (FITC) (clone HI30; eBioscience, San Diego, CA, USA), PD-1-Phycoerythrin (PE)/DazzleTM 594 (clone EH12.2H7; BioLegend), LAG-3-Brilliant violet 421 (clone T47-530; BD Biosciences), and TIM-3-Brilliant Violet 711 (clone 7D3; BD Biosciences). Stained cells were incubated at 4 °C for 30 min and analyzed by flow cytometry.

Intracellular staining was also performed as previously described [16]. Briefly, cells were incubated in a fixation/permeabilization buffer (eBioscience) and then washed twice with a permeabilization wash buffer (eBioscience). Rat serum (Sigma-Aldrich) and mouse serum (Sigma-Aldrich) were used to block non-specific binding sites. Intracellular antibodies against FoxP3-phycoerythrin cyanin 7 (PE/Cy7) (clone PCH101; eBioscience) and Helios-FITC (clone 22F6; BioLegend) were added, and cells were incubated for 30 min at 4 °C. Cells were washed twice with a permeabilization wash buffer (eBioscience), and re-suspended in a flow cytometry staining buffer.

All data were acquired by a BD LSRFortessa X-20 SORP flow cytometer (BD Biosciences) operating on the BD FACSDiva software (BD Biosciences), and they were analyzed with the FlowJo V10 software (FlowJo, Ashland, OR, USA).

### 2.4. Library Preparation and RNA Sequencing

Total RNA was extracted from the cell suspensions of the explant culture, which contained a heterogeneous population of tumor-infiltrating immune cells, using a DNA/RNA/Protein Purification Plus Micro Kit (Norgen, Thorold, ON, Canada). RNA concentrations were determined by Qubit RNA HS (Invitrogen). The cDNA libraries were generated using TruSeq RNA Library Prep Kit (Illumina, San Diego, CA, USA) following the manufacturer’s instructions. The quality of libraries was checked by Agilent High Sensitivity DNA Kit (Agilent Technologies, Santa Clara, CA, USA), and quality-passed DNA (>2000 bp) were quantified and processed, as previously described [23]. Libraries that passed the quality control were clustered and sequenced on an Illumina HiSeq 4000 instrument using HiSeq 3000/4000 SBS kit (Illumina).

### 2.5. RNA Sequencing Data Analyses

Quality-trimmed pair end reads were aligned to the hg19 human reference genome in CLC Genomics Workbench-12 (Qiagen) [17,24]. The abundance of the expression of transcripts was determined as the score of TPM (transcripts per million) mapped reads. Abundance data were subsequently subjected to differential gene expression using built-in statistical analyses recommended in CLC Genomics protocol with 2-fold change and a *p* value cutoff of <0.05. For heatmaps, Z-scores (as previously described [25]) were calculated from TPM values for differentially expressed genes with *p* values of <0.05 from treated and non-treated cells. Data shown in the heatmaps represent the mean Z-score for each gene obtained from two independent samples (patients #57 and 59) for each treatment group.

### 2.6. Functional Annotation Analyses Using DAVID Platform

The “gene ontology biological process (GO BP),” “Kyoto Encyclopedia of Genes and Genomes (KEGG),” and “BioCarta” network analyses [26,27] were performed on the Database for Annotation, Visualization and Integrated Discovery (DAVID) platform (v.6.8, https://david.ncifcrf.gov), as previously described [23]. We uploaded the list of upregulated and downregulated genes (with a *p* value cutoff of <0.05) separately on the DAVID platform to obtain functional annotations. The data from functional analyses are presented as heatmaps. For pathway enrichment analysis and bar plots, the Z-score for each pathway was calculated as the mean of fold change of individual genes (comparing treated cells with non-treated cells) enriched within that pathway, as previously described [25].

## 3. Results

### 3.1. Effects of Immune Checkpoint Inhibition on Ex Vivo Expanded Tumor-Infiltrating T Cells

Multiple ICs are expressed on activated T cells, but excessive stimulation during in vitro expansion may lead to T cell exhaustion, which is characterized by the overexpression of inhibitory ICs [28]. Previously, we showed that the co-blockade of PD-1 and PD-L1 upregulated the surface expression of CTLA-4, TIM-3, and LAG-3 on CD4^+^ T cell subsets by using a co-culture system with human breast cancer cell lines [20].

**Figure 1:**
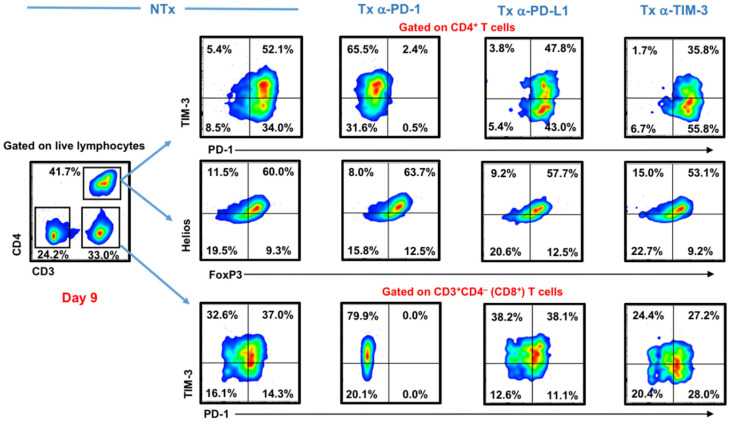
Effect of different immune checkpoint inhibitors on T cells in breast tumor explants. Tumor tissue from 2 breast cancer patients were cut into small pieces and cultured with exogenous interleukin-2 (IL-2), in the presence or absence of anti-programmed cell death protein 1 (PD-1), anti-programmed death ligand-1 (PD-L1), or anti-T cell immunoglobulin and mucin-domain containing-3 (TIM-3) monoclonal antibodies (mAbs). Cells were collected on Day 9 and stained with TIM-3, PD-1, and different T regulatory cell (Treg)-related markers. Representative flow cytometric plots show TIM-3 and PD-1 surface expression on CD3^+^CD4^−^ (CD8^+^) and CD3^+^CD4^+^ T cells, as well as intracellular FoxP3 and Helios expression on CD3^+^CD4^+^ T cells from different treatment conditions.

Here, we investigated the effects of the different ICIs on expanded T cell populations (both CD4^+^ and CD8^+^ T cells) after nine days in human breast tumor explant culture. We maintained explant cultures of breast tumor tissues and investigated the immune phenotypes of expanded T cell populations in the presence or absence of different ICIs. We found that TIM-3 and PD-1 were expressed at high levels on expanded CD4^+^ and CD8^+^ T cells in the non-treated control (Figure 1). The PD-1 blockade completely diminished PD-1 surface expression on T cells, while the PD-L1 blockade did not affect PD-1 or TIM-3 surface expression on both T cell populations. The TIM-3 blockade reduced TIM-3 surface expression on both CD4^+^ and CD8^+^ T cells (Figure 1).

We also investigated the effects of different ICIs on expanded FoxP3^+^ Tregs. Tregs are known to be a key component of the immunosuppressive profile of the TME of various cancers, and their levels are frequently associated with disease progression [29]. We found that CD4^+^FoxP3^+^ Tregs were expanded in all conditions, and they also co-expressed Helios, an important transcription factor associated with stability and function of Tregs [30]. Importantly, we found that none of the ICIs affected the levels of CD4^+^FoxP3^+^Helios^+/−^ Tregs (Figure 1).

### 3.2. Genes Associated with Immune Response, IFN-γ-Mediated Pathway, Activation MAPK Signaling and Apoptosis Were Upregulated in Pembrolizumab-Treated Cells

**Figure 2:**
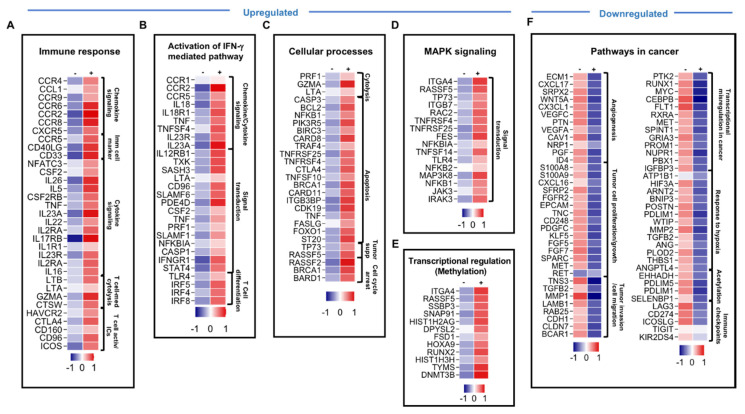
Differential gene expression of breast tumor-infiltrating immune cells in response to pembrolizumab treatment. Heatmaps show the Z-score calculated from the transcript per million (TPM) of each gene to compare the expression level in tumor-infiltrating immune cells treated with anti-PD-1 vs. non-treated cells. Each column represents a sample pooled from two explant cultures either treated or untreated, and each row represents the Z-score for mean expression obtained from two explant cultures (patients #57 and 59). The Z-score for mean expression level of each gene is depicted according to color scale. The functional categorization of top significantly upregulated and downregulated genes (with a fold change of >2 and *p* value <0.05 cutoffs) from CLC analysis were analyzed separately through DAVID platform. Genes involved in immune response (**A**), the activation of the IFN-γ-mediated signaling pathway (**B**), cellular processes (**C**), MAPK signaling (**D**), and genes related to transcriptional regulation via methylation (**E**) were upregulated in response to anti-PD-1, while genes in cancer-related pathways (**F**) were downregulated.

In order to investigate the effects of different ICIs on TIICs at the transcriptomic level, we harvested the cells after 25 days in culture, extracted RNA, and performed RNA-Seq on non-treated cells and those treated with different ICIs. Culturing peripheral blood mononuclear cells (PMBCs) or tumor explants in the presence of exogenous IL-2 results in the survival and selective enrichment of T cells, so we referred to them in a general term as “tumor-infiltrating immune cells.” A total of 750 upregulated and 1073 downregulated transcripts were identified in TIICs treated with pembrolizumab, compared with the non-treated TIICs. Only genes and pathways that were significantly affected, with a fold change >2 and a *p* value cutoff of <0.05, were selected for further analysis. Genes from RNA-Seq data were identified and classified into functional categories using GeneCards and the DAVID. RNA-Seq results showed that upregulated genes in TIICs treated with pembrolizumab were related to immune response, such as chemokines and chemokine receptors (CCL1, CCR2, CCR6, CCR8, and CXCR5), immune cell markers (CD33, CD40LG and NFATC3), cytokines (CSF2, IL23A, TNF, IL22, and IL26) and cytokine receptors (CSF2RB, IL23R, IL2RA, IL17RB, and IL1R) (Figure 2A), as well as T cell activation markers/immune checkpoints (HAVCR2, CTLA4, CD160, ICOS, and CD96) were upregulated upon pembrolizumab treatment (Figure 2A and Table 1). Notably, genes related to the activation of IFN-γ-mediated signaling pathway, such as CCR2, CSF2, IL23A, IL23R, TLR4, TXK, STAT4, TNF, IRF8, IRF4, and IRF5, were upregulated upon pembrolizumab treatment (Figure 2B). Genes related to T cell cytolytic activity, such as GZMA gene-encoding granzyme A, PRF1 gene-encoding perforin, and LTA gene-encoding lymphotoxin-α, were upregulated upon anti-PD-1 treatment (Figure 2C). In addition, genes promoting cell apoptosis, such as TRAF4, TNFSF10, CTLA4, BCL2, BIRC3, CDK19, CARD11, TNFRSF25, TNFRSF8, TNF, and TNFSF11A; tumor suppressor genes, such as TP73 and ST20; genes involved in DNA repair and cell cycle arrest, such as BRCA1, RASSF2, and BARD1, were also upregulated following PD-1 blockade (Figure 2C). Genes in the MAPK signaling pathway, such as ITGA4, RASSF5, TNFRSF4, TP73, JAK3, IRAK2, IRAK3, RAC2, RASA2, MAP3K8, ITGB7, TLR4, and NFKB2 (Figure 2D), in addition to genes related to transcriptional regulation via methylation, such as DNMT3B and HIST1H3H were also upregulated upon anti-PD-1 mAb (Figure 2D,E). Collectively, these findings indicated that anti-PD-1 mAbs upregulate genes that can enhance anti-tumor immunity and induce tumor suppression/apoptosis. These upregulated genes are involved in immune cell proliferation (via MAPK signaling pathway [31]), immune cell activation and migration via cytokines and chemokines [32], tumor cell cytotoxicity [33], and tumor suppression/apoptosis to control tumor growth.

### 3.3. Genes Associated with Cancer-Related Pathways and Acetylation, and Genes Encoding Immune Checkpoints Were Downregulated in Pembrolizumab-Treated Cells

We found that genes associated with cancer development and acetylation were downregulated in pembrolizumab-treated TIICs. Genes related to cancer pathways were downregulated upon pembrolizumab treatment, including angiogenic factors for tumor vascularization (NRP2, PTN, VEGFC, ID4 and ESM1), proto-oncogenes (RET and MET), tumor cell growth and proliferation genes (SPARC, FGFR2, TNC, KLF5, EGFR, PDGFC, FGF5, FGF7, KLF5, S100A8, and S100A9) and tumor migration/invasion-related genes (CDH1, CLDN7, TNC, BCAR1, MMP12, TGFB2, TNS3, LAMB1, MMP1, and RAB25) (Figure 2F). Some of these genes, which interfere with tumor cell growth/differentiation/migration/invasion (such as MYC, MET, PROM1, and PTK2), induce hypoxia (such as ARNT2, HIF3A, TGB2, MMP2, and POSTN) and genes regulating transcription via acetylation were downregulated upon pembrolizumab treatment (Figure 2F). Additionally, we found that pembrolizumab reduced the expression of multiple ICs/IC ligands, including LAG3, CD274, ICOSLG, LGALS9, TIGIT and KIR2DS4 (Figure 2F and Table 1). Overall, these results indicated that anti-PD-1 mAb could suppress tumor angiogenesis, growth, invasion, and metastasis, and may interfere with gene transcription by inhibiting acetylation and regulating the expression of other IC/IC ligand expression.

### 3.4. Atezolizumab Upregulated the Expression of Different Genes Involved in Immune Response, IFN-γ-Mediated Pathway, Apoptosis and MAPK Signaling, and Downregulated Genes Involved in Cancer-Related Pathways and Hippo Signaling Pathway

**Figure 3:**
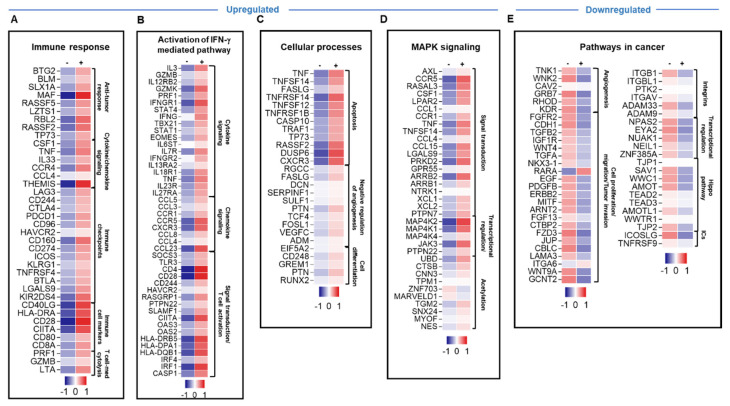
Differential gene expression of breast tumor-infiltrating immune cells in response to atezolizumab treatment. Heatmaps show the Z-score calculated from the TPM of each gene to compare the expression level in tumor-infiltrating immune cells treated with anti-PD-L1 vs. non-treated cells. Each column represents a sample pooled from two explant cultures either treated or untreated, and each row represents the Z-score for mean expression obtained from two explant cultures (patients #57 and 59). The Z-score for the mean expression level of each gene is depicted according to color scale. The functional categorization of top significantly upregulated and downregulated genes (with a fold change of >2 and *p* value <0.05 cutoffs) from CLC analysis were separately analyzed through the DAVID platform. Genes involved in immune response (**A**), the activation of the IFN-γ-mediated signaling pathway (**B**), cellular processes (**C**), and MAPK signaling (**D**) were upregulated in response to anti-PD-L1, while genes in cancer-related pathways (**E**) were downregulated.

We then compared atezolizumab-treated TIICs with non-treated cells. A total of 1700 upregulated and 579 downregulated transcripts were identified in TIICs treated with atezolizumab. Only genes and pathways that were significantly affected, with a fold change of >2 and a *p* value cutoff of <0.05, were selected for further analysis. Atezolizumab upregulated genes related to immune response, such as cytokines (TNF, CSF1, and IL33), chemokines (THEMIS and CCL4), immune cell markers, co-stimulatory and antigen presentation related molecules (CD8A, CD40LG, CD28, CD80, CIITA and HLA-DRA), and T cell cytolytic activity (PRF1, GZMB and LTA) were upregulated following atezolizumab treatment (Figure 3A). Additionally, genes related to T cell activation markers and immune checkpoints (LAG3, CD244, CTLA4, PDCD1, CD96, HAVCR2, CD160, ICOS, CD274, KLRG1, BTLA, KIR2DS4, TNFRSF4, and LGALS9) were upregulated following atezolizumab treatment (Figure 3A and Table 1). Genes related to the activation of IFN-γ-mediated signaling pathway, such as IFNG, TBX21, STAT1, STAT4, IFNGR1, EOMES, TNF, CCL4, TLR3, IRF4, and HLA-DRB5, were also upregulated upon atezolizumab treatment (Figure 3B). In addition, tumor suppressor genes (such as TP73) and genes related to cell apoptosis and cell cycle arrest (such as CARD17, TRAF1, CASP10, FASLG, TNFSF14, TNFRSF12, TNF, TNFRSF1B, and RASSF2) were upregulated following atezolizumab treatment (Figure 3C). Genes associated with cell differentiation were also upregulated following drug treatment (Figure 3C). Moreover, atezolizumab upregulated genes associated with MAPK signaling pathway, such as TNF, CCR1, and MAPK4K2, and genes associated with acetylation, such as UBD and CTSB (Figure 3D).

Genes related to cancer pathways were downregulated upon atezolizumab treatment, including angiogenic factors for tumor vascularization (TNK1, AREG and KDR), proto-oncogenes (RET and MET) and tumor cell survival/migration/invasion (CDH1, RARA, WNK2, WNT4A, WNT9A, TGFB2, EGF, and LAMA3) (Figure 3E). In addition, genes regulating transcription via acetylation (NES, TGM2 and MYOF) were downregulated upon atezolizumab treatment (Figure 3E). Genes encoding for integrins (such as ITGAV, ITGBL1, and ITGB1) that can promote tumor cell proliferation and migration [34] and genes related to the Hippo signaling pathway, one of the signaling pathways which has been implicated in cancer by promoting cell proliferation [35], were also downregulated (Figure 3E). Additionally, we found that genes encoding other IC ligands, such as ICOSLG and TNFRSF9, were downregulated upon atezolizumab treatment (Figure 3E and Table 1). Taken together, these results indicated that anti-PD-L1 mAb can promote anti-tumor immunity, antigen presentation, T cell activation, and T cell cytotoxicity, and can suppress tumor angiogenesis, growth, invasion, and metastasis.

### 3.5. TIM-3 Blockade Upregulated Genes of Immune Response-, IFN-γ-Mediated Pathway-, Apoptosis, MAPK Signaling- and Acetylation-Related Genes, but Downregulated Genes Involved in Cancer-Related Pathways and JAK–STAT Pathway

**Figure 4:**
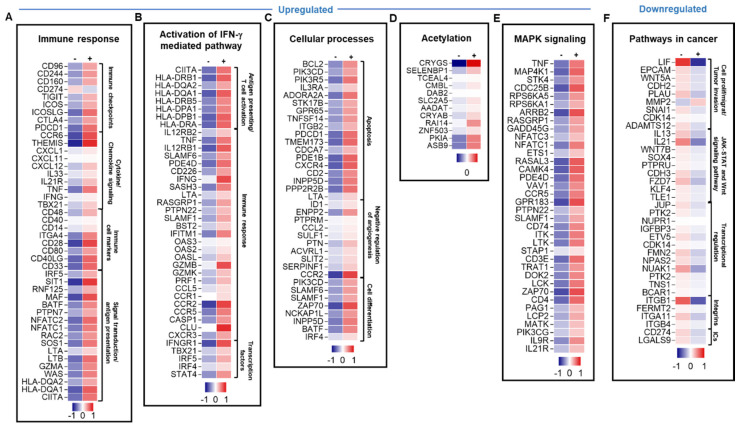
Differential gene expression of breast tumor-infiltrating immune cells in response to anti-TIM-3 mAb treatment. Heatmaps show the Z-score calculated from TPM of each gene to compare the expression level in tumor-infiltrating immune cells treated with anti-TIM-3 vs. non-treated cells. Each column represents a sample pooled from two explant cultures either treated or untreated, and each row represents the Z-score for mean expression obtained from two explant cultures (patients #57 and 59). The Z-score for mean expression level of each gene is depicted according to color scale. The functional categorization of top significantly upregulated and downregulated genes (with a fold change of >2 and *p* value <0.05 cutoffs) from CLC analysis were analyzed separately through the DAVID platform. Genes involved in immune response (**A**), the activation of the IFN-γ-mediated signaling pathway (**B**), cellular processes (**C**), MAPK signaling (**D**), and acetylation (**E**) were upregulated in response to anti-TIM-3, while genes in cancer-related pathways (**F**) were downregulated.

TIM-3 blockade resulted in the upregulation and downregulation of different sets of genes associated with different pathways. A total of 1073 upregulated and 560 downregulated transcripts were identified in TIICs treated with anti-TIM-3 mAb. Only genes and pathways that were significantly affected, with a fold change of >2 and a *p* value cutoff of <0.05, were selected for further analysis. Genes related to immune response, such as cytokines (TNF, IL33, and IFNG), cytokine receptors (IL12R), chemokines (THEMIS, CXCL1, CXCL11, and CXCL12), immune cell markers co-stimulatory receptors (CD40, CD40LG, CD33, CD28, and CD80), and other genes related to signal transduction and antigen presentation were upregulated following TIM-3 blockade (Figure 4A). ICs/IC ligands, such as ICOS, ICOSLG, PDCD1, TIGIT, CTLA4, CD96, CD160, CD244, KLRG1, TNFRSF4, BTLA, CD274, TNFRSF9, and KIR2DS4, were upregulated upon anti-TIM-3 mAb treatment (Figure 4A and Table 1). Furthermore, genes related to the activation of IFN-γ-mediated signaling pathway, such as CD226, TNF, IFNG, IFNGR1, STAT4, IRF4, IRF5, TBX21, SLAMF1, SLAMF6, LTA, IFITM1 and CIITA, were upregulated upon anti-TIM-3 mAb treatment (Figure 4B). Genes promoting cell apoptosis and genes that negatively regulate angiogenesis were upregulated upon anti-TIM-3 mAb treatment (Figure 4C). Genes related to cell differentiation were also upregulated following anti-TIM-3 mAb treatment (Figure 4C). Genes regulating transcription via acetylation were upregulated upon anti-TIM-3 mAb treatment (Figure 4D). Anti-TIM-3 mAb treatment upregulated genes related to the MAPK signaling pathway (Figure 4E).

The blockade of TIM-3 in breast tumor explants downregulated genes related to cancer pathways, including those associated with tumor cell proliferation/migration/invasion (WNT5A, LIF, XDH2, SNAI, CDH2, ADAMST12, PLAU, and CDK14) (Figure 4F). In addition, genes related to JAK–STAT and Wnt signaling pathways, transcriptional regulations for tumor promoting genes, and integrins were also downregulated upon TIM-3 blockade (Figure 4F). IC ligands such as CD274 and LGALS9 were downregulated upon anti-TIM-3 mAb treatment (Figure 4F and Table 1).

Collectively, these data showed that anti-TIM-3 mAb can enhance anti-tumor immunity by upregulating genes that favor immune cell proliferation/activation and T cell cytotoxicity, and it can also suppress tumor angiogenesis, growth, invasion, and metastasis by downregulating genes associated with the JAK–STAT and Wnt signaling pathways, and integrins [34,36,37].

### 3.6. Pembrolizumab, Atezolizumab and TIM-3 Blockade Can Regulate Distinct Molecular Pathways in Breast Cancer Explants

**Figure 5:**
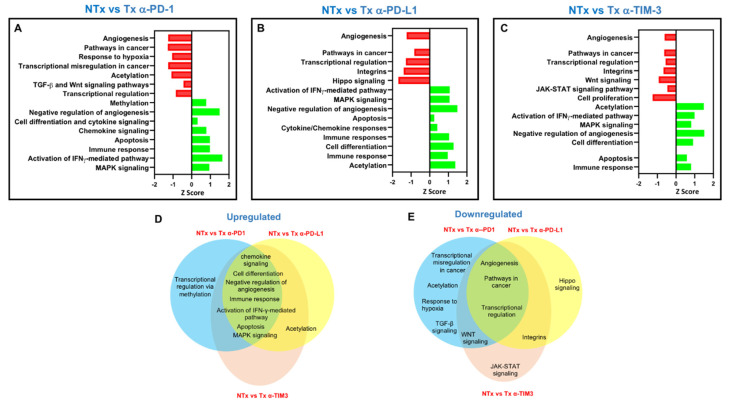
Analyses of overlapping functional pathways between the response of breast tumor-infiltrating immune cells to PD-1, PD-L1 or TIM-3 inhibition. The horizontal bars denote the top significantly affected pathways (with a fold of change of >2 and a *p* value cutoff of <0.05) based on the Kyoto Encyclopedia of Genes and Genomes (KEGG) pathway and DAVID functional annotation analyses for NT (non-treated cells) vs. anti-PD-1 (**A**), NT vs. anti-PD-L1 (**B**), and NT vs. anti-TIM-3 (**C**). Venn diagram summarizing the overlap between functional pathways that were upregulated (**D**) and downregulated (**E**) in the comparative analyses between non-treated and treated cells with either of the immune checkpoint inhibitors (ICIs). Shared pathways are indicated by the overlap between circles.

Our overall transcriptomic data revealed some distinct mechanisms for the three investigated ICIs. The effect of pembrolizumab on TIICs is related to several functional networks and signaling pathways (Figure 5A). Pathways implicated in transcriptional regulation, TGF-β and Wnt signaling, acetylation, cancer related-pathways, angiogenesis, and cell differentiation were downregulated upon pembrolizumab treatment (−1.5 > Z-score < −0.5; Figure 5A). On the other hand, pathways related to methylation, chemokine signaling, apoptosis, cytokine signaling, immune response, negative regulation of angiogenesis, the activation of the IFN-mediated pathway, and MAPK signaling were upregulated (0.4 > Z-score < 1.9; Figure 5A).

Atezolizumab downregulated pathways related to transcriptional regulation, Hippo signaling, acetylation, cancer related-pathways, angiogenesis, and integrins (−1.9 > Z-score < −0.5, Figure 5B). On the other hand, pathways related to anti-tumor responses, cell differentiation, apoptosis, chemokine/cytokine responses, immune response, the negative regulation of angiogenesis, the activation of the IFN-γ-mediated pathway, and MAPK signaling were upregulated (0.2 > Z-score < 1.9, Figure 5B).

Anti-TIM-3 downregulated pathways related to transcriptional regulation, integrins, cell proliferation, cancer related-pathways, JAK–STAT signaling, angiogenesis, the negative regulation of apoptosis, and Wnt signaling (−1.3 > Z-score < −0.2, Figure 5C). On the other hand, pathways related to acetylation, cell differentiation, apoptosis, TGF-β signaling, immune response, the negative regulation of angiogenesis, the activation of the IFN-γ-mediated pathway, and MAPK signaling were upregulated (0.7 > Z-score < 1.9; Figure 5C).

Venn diagrams showed that targeting PD-1, PD-L1, and TIM-3 in breast cancer explants resulted in the upregulation or downregulation of common and distinct signaling pathways (Figure 5D,E). Targeting PD-1 exclusively upregulated pathways related to methylation and downregulated pathways related to acetylation, response to hypoxia, and TGF-β signaling. On the other hand, targeting PD-L1 exclusively downregulated genes related to the Hippo signaling pathway. The blockade of TIM-3 exclusively downregulated genes related to the JAK–STAT signaling pathway.

Altogether, these data indicated that the three inhibitors seem to interfere with cancer-related pathways to exert anti-tumorigenic effects and enhance anti-tumor immunity. This could occur by favoring T cell activation and cytolytic activity, and by suppressing signaling pathways favoring tumor cell proliferation, migration, and tumor invasion/metastasis.

## 4. Discussion

In this study, we performed phenotypical characterizations of the expanded T cell populations, in addition to transcriptomic analyses on cells from the different ICI treatment conditions. to investigate the molecular pathways and the biological mechanisms they regulate in PBC. We found that tumor-infiltrating T cells were expanded in the presence of exogenous IL-2 with upregulated surface expression of TIM-3 and PD-1. The use of exogenous IL-2 was necessary for our explant culture to maintain the survival and proliferation of TILs [38]. However, this in vitro expansion of T cells led to differentiated phenotypes and the heterogeneity of the expanded populations [39].

Unlike anti-TIM-3 mAb, pembrolizumab treatment resulted in a complete blockade of the PD-1 surface expression on T cell subsets; this complete blockade was most likely due to a “masking effect” blocking the epitope of PD-1 by pembrolizumab, so the fluorescence-activated cell sorting (FACS) detection antibody would not be able to bind the same epitope of PD-1 showing no expression. This justification was supported and confirmed by qPCR and Western plot data indicating that pembrolizumab does not alter the expression of PD-1 at the mRNA and protein levels, as it only blocks its epitope [17].

Immunosuppressive cells, such as Tregs, in the TME are associated with resistance to immunotherapy [40,41]. Interestingly, the presence of FoxP3^+^ Tregs in breast cancer patients has been associated with recurrence-free survival [42]. Therefore, we investigated the effects of different ICIs on tumor-infiltrating FoxP3^+^ Tregs in breast cancer patients. We have previously reported that anti-PD-1 (pembrolizumab) does not affect the phenotype or function of Tregs, as it instead affects the differentiation of FoxP3^+^-induced Tregs [17,18]. In the present study, we found that the levels of FoxP3^+^Helios^+^ Treg remained unaffected upon the treatment with anti-PD-1. Importantly, in this study, we found that the TIM-3 and PD-L1 blockades also did not affect the levels of FoxP3^+^Helios^+^ Treg, thereby suggesting that ICIs do not modulate the functionality of Tregs. However, it is noteworthy that we investigated the effects of ICIs on both natural and induced Treg, and we did not probe their functional characteristics. FoxP3 can be induced on naïve T cells via antigenic stimulation, but these cells may not have any immunosuppressive potentials [43].

Previously, we reported the effects of blocking PD-L1 in human TNBC cell line, MDA-231, and we elucidated signaling and functional pathways that were altered in response to atezolizumab [19]. Here, we investigated the mechanisms and signaling pathways that were affected upon the blockade of PD-1, PD-L1 or TIM-3, in TIICs isolated from breast cancer explants.

The density and type of TILs, along with the type of soluble factors (e.g., growth factors and cytokines) present within the TME reflect the ability of the immune response to eradicate tumor cells and dictate the response to therapy in cancer patients, including those with breast cancer [44,45]. In the majority of cancer types, including breast cancer, improved prognosis and favorable clinical outcomes in patients have been associated with increased levels of CD8^+^ TILs and Th1 response [45,46,47]. Reduced levels of genes encoding inflammatory cytokines and genes related to Th1 response have been detected in breast tumor tissues, particularly in advanced stages, compared to normal tissues, suggesting the importance of these genes in regulating tumorigenesis [47]. The response of breast cancer explants upon PD-1, PD-L1, or TIM-3 inhibition was also indicated by the increased expression of genes related to the activation of anti-tumor immune responses, including inflammatory cytokines, such as TNF, GM-CSF (CSF2 gene), IFN-γ, chemokines, cytolytic molecules (such as perforin and granzymes), receptors associated with antigen presentations, immune cell markers, and co-stimulatory molecules (such as HLADR, CD8A, CD4, CD40LG, CD80 and CD28) [48,49]. The blockade of PD-1, PD-L1, or TIM-3 resulted in the upregulation of various genes related to the activation of the IFN-γ signaling pathway; some of them encode inflammatory cytokines (such as IFN-γ, TNF-α, and GM-CSF), transcription factors (such as T-bet) and key signaling mediators (such as STAT1 and STAT4) involved in the maintenance and differentiation of the Th1 immune response, which plays a key role in anti-tumor immunity, and the recruitment and activation of effector immune cells, such as M1 macrophages, dendritic cells and natural killer (NK) cells [45,46,50]. In addition, genes encoding transcription factors related to CD8^+^ effector T cell and dendritic cell differentiation, such as IRF4, IRF5, and IRF8 [51,52,53], were upregulated in response to the different ICI treatments. The blockade of PD-L1 and TIM-3 resulted in the downregulation of integrins, which can significantly contribute to tumor cell proliferation and migration [34]. These findings may suggest that the blockade of these ICIs can promote antigen presentation, and T cell activation, along with other effector immune cells, enhances CD8^+^ and CD4^+^ Teff functions and leads to tumor cytotoxicity.

Although the response to each ICI showed common pathways, their individual blockades resulted in the upregulation of distinct sets of genes. We found that the inhibition of PD-1 upregulated the gene expression of other co-inhibitory and co-stimulatory ICs, including TIM-3 (gene HAVCR2), CTLA4, CD96, ICOS, and CD160, while the blockade of PD-L1 upregulated the expression of LAG3, PDCD1 (PD-1 gene), CTLA4, CD244, CD96, HAVCR2, CD160, ICOS, CD274, KLRG1, BTLA, KIR2DS4, TNFRSF4 (OX40 ligand), and LGALS9 (Figure 2A and Figure 3A; Table 1). The inhibition of TIM-3 upregulated the expression of co-stimulatory ICs, ICOS, ICOSLG, TNFRSF4, TNFRSF9, and KIR2DS4, which activated CD8^+^ effector T cell function and CD4^+^ Th1 cells [54,55], as well as co-inhibitory ICs, PDCD1, TIGIT, CD96, CD244, CD160, KLRG1, BTLA, CD274, and CTLA-4 (Figure 4A and Table 1). Together, these data suggested that blocking one IC or IC ligand can result in the upregulation of alternative ICs, which potentially leads to T cell exhaustion [56] and gives rise to compensatory mechanisms for tumor cell survival and immune response evasion leading to acquired resistance [20,41]. In parallel with this, we previously investigated the effect of blocking PD-1 and PD-L1 on CD4^+^ T cells, including Tregs, using a co-culture model of the MDA231 human TNBC cell line [20]. We found that the single or dual blockade of PD-1 and PD-L1 resulted in the upregulation of other ICs on CD4^+^ T cells, such as TIM-3, CTLA-4 and LAG-3, indicating the emergence of compensatory mechanisms that potentially lead to resistance to ICIs [20]. Other studies have also shown that resistance-mediated mechanisms in response to PD-1 or PD-L1 blockades are associated with the overexpression of alternative ICs in murine and human studies [57,58,59,60].

In addition, IFNG, TBX21 (gene-encoding T-bet, a major transcription factor in Th1 response and IFN-γ signaling) and STAT1 (a key signaling mediator in the Th1 response and IFN-γ signaling) [50] were significantly upregulated in response to anti-PD-L1 (Figure 3B), and TBX21 and IFNG genes were upregulated in response to anti-TIM-3 (Figure 4B), compared to non-treated cells. However, these genes were not significantly affected in response to anti-PD-1, suggesting that different treatments can affect a different set of genes.

Cancer-related pathways that promote tumor growth, angiogenesis, invasion, and metastasis can be driven by many signaling cascades, such as those mediated by MAPK, PI3K/Akt, Wnt, Notch, and Hippo signaling pathways [61,62]. Multiple pathways were commonly upregulated upon the inhibition of PD-1, PD-L1, or TIM-3, associated with the MAPK signaling pathway, cell differentiation, cell apoptosis, cytolysis, tumor suppression, cell cycle arrest and anti-angiogenesis,. It has been reported that the MAPK pathway or some of its components, e.g., p38 MAPK, can play a dual role in cancer; they can either promote or inhibit tumor survival/growth depending on the cell type or stimulus [63]. Studies have shown that the activation of the p38 MAPK pathway can inhibit mammary tumorigenesis through the induction of p53-mediated apoptosis [63,64,65], implicating its role in tumor suppression (Supplementary Figure S1). Apart from this, other components of MAPK signaling pathways, namely ERK, has been implicated in the regulation of CD8^+^ T cell proliferation and survival [31].

Although the single blockades of PD-1, PD-L1, and TIM-3 commonly downregulated pathways involved in tumorigenesis, angiogenesis, and transcriptional regulation, the response of breast cancer explants to each ICI showed distinct signaling pathways that were affected upon the inhibition of one particular IC. We found that PD-1 blockade was able to upregulate pathways associated with methylation and downregulate genes associated with acetylation, response to hypoxia, and TGF-β signaling. Hypoxic conditions are toxic for both normal and tumor cells; however, tumor cells can overcome this by acquiring epigenetic modifications (e.g., DNA methylation and histone methylation/acetylation) to promote their survival and growth in such conditions via the activation of the hypoxia-inducible factor (HIF) pathway [66,67]. Additionally, the activation of the HIF pathway can also induce epithelial-mesenchymal transition (EMT) and favor tumor cell migration, invasion, and metastasis [68]. Together, these findings suggested that PD-1 blockade in PBC explant cultures could negatively regulate the hypoxic response by increasing methylation and reducing acetylation, thus causing gene transcriptional silencing and the deactivation of TGF-β signaling, and leading to reduced tumor cell proliferation, growth, and invasion.

The relationship between the Hippo pathway, one of the key pathways implicated in cancer and the promotion of tumor cell proliferation and growth [35], and PD-L1 has been previously reported. Rensburg et al. demonstrated that the Hippo signaling pathway supports tumor growth and immunosuppression by inducing the expression of PD-L1 [69]. Here, we found that the inhibition of PD-L1, amongst other ICIs, exclusively downregulated genes related to the Hippo pathway. Together, these data implicate the mutual relationship between PD-L1 and the Hippo pathway in cancer.

The JAK–STAT signaling pathway is known to act as double-edged sword in cancer; it can be beneficial or deleterious depending on the STATs activated [36]. Of note, we found that TIM-3 inhibition downregulated JAK–STAT pathway-related genes that potentially comprise the “bad STATs” associated with tumorigenesis and immunosuppression. In support of this, we found that TIM-3 inhibition increased the expression of genes associated with increased antigen-presentation, enhancement of T cell cytotoxicity, and increased expression of pro-inflammatory cytokines (Figure 5).

## 5. Conclusions

In conclusion, our transcriptomic data revealed some of the unique mechanisms of action of mAbs targeting PD-1, PD-L1, and TIM-3 in human breast cancer explants. These findings furthered our understanding of the signaling pathways regulated by these ICIs in breast cancer. However, further investigations in a larger number of samples and functional studies are warranted to validate these findings. In addition, this work could be extended to investigate the effect of other ICIs, such as anti-CTLA-4, anti-LAG-3, and anti-TIGIT.

## Figures and Tables

**Table 1:** Differentially expressed immune checkpoints (receptors and ligands) in tumor-infiltrating immune cells from breast tumor explants in response to anti-PD-1, PD-L1, or anti-TIM-3 compared to non-treated cells.

Immune Checkpoints	Anti-PD-1	Anti-PDL1	Anti-TIM-3
Upregulated	HAVCR2 (TIM-3 gene), CTLA4, CD96, ICOS and CD160	LAG3, PDCD1 (PD-1 gene), CTLA4, CD244, CD96, HAVCR2, CD160, ICOS, CD274, KLRG1, BTLA, KIR2DS4, TNFRSF4 (OX40 ligand) and LGALS9	ICOS, ICOSLG, PDCD1, TIGIT, CTLA4, CD96, CD160, CD244, KLRG1, TNFRSF4, BTLA, CD274, TNFRSF9 and KIR2DS4
Downregulated	LAG3, CD274 (PD-L1), ICOSLG (ICOS ligand), TIGIT, LGALS9 (galectin-9) and KIR2DS4	ICOSLG and TNFRSF9 (CD137)	CD274 and LGALS9

## Supplementary Materials

The following are available online at https://www.mdpi.com/2073-4425/11/6/703/s1, Figure S1: Activation of the p38 MAPK pathway could inhibit mammary tumorigenesis through the induction of p53-mediated apoptosis. Data Availability: RNA-Seq raw data were deposited in NCBI SRA, with BioProject ID PRJNA639313.
