# Supplementary material for: Blockade of PD-1, PD-L1, and TIM-3 Altered Distinct Immune- and Cancer-Related Signaling Pathways in the Transcriptome of Human Breast Cancer Explants
Source: Genes (Basel). 2020 Jun 25;11(6):703. doi: 10.3390/genes11060703 (PMC7349021; doi:10.3390/genes11060703)
Supplement: Supplementary file 1 [file genes-11-00703-s001.pdf]

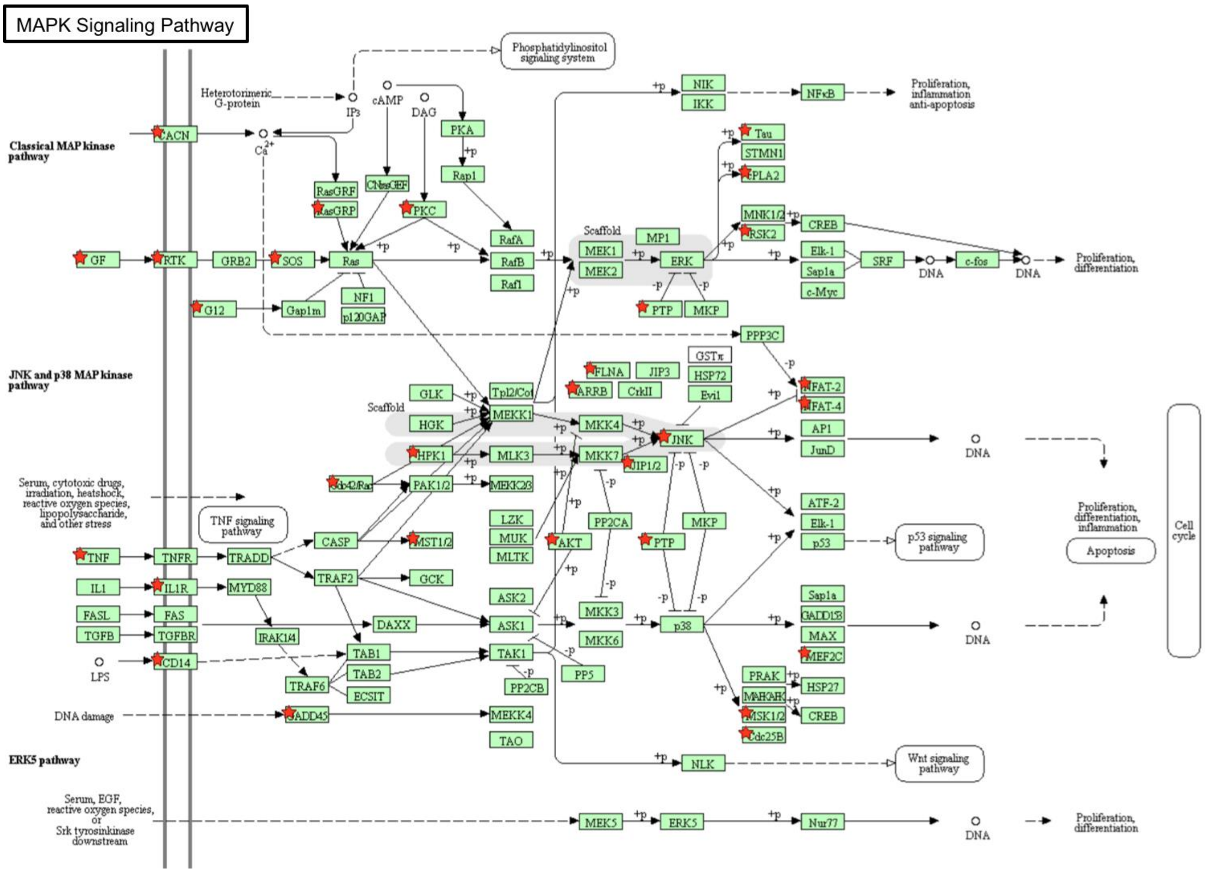

Supplementary Figure 1. Activation of the p38 MAPK pathway could inhibit mammary tumorigenesis through the induction of p53-mediated apoptosis. Proliferation, differentiation and inflammation targeted genes (red star) are involved in the MAPK signaling pathway were upregulated in all the treatment groups.
